# Technical considerations of endovascular management of true visceral artery aneurysms

**DOI:** 10.1186/s42155-023-00368-9

**Published:** 2023-06-07

**Authors:** M. K. Khairallah, R. A. Morgan, R. Das

**Affiliations:** 1 Department of Radiology, St.George’s University Hospitals NHS Foundation Trust, Blackshaw Road, London, SW17 0QT UK; 2 Assiut University Hospital, Assiut, Egypt

**Keywords:** Visceral aneurysm, Arterial embolisation, Renal, Mesenteric

## Abstract

**Background:**

True visceral artery aneurysms are potentially complex to treat but with advances in technology and increasing interventional radiology expertise over the past decade are now increasingly the domain of the interventional radiologist.

**Body:**

The interventional approach is based on localization of the aneurysm and identification of the anatomical determinants to treat these lesions to prevent aneurysm rupture. Several different endovascular techniques are available and should be selected carefully, dependent on the aneurysm morphology. Standard endovascular treatment options include stent-graft placement and trans-arterial embolisation.

Different strategies are divided into parent artery preservation and parent artery sacrifice techniques.

Endovascular device innovations now include multilayer flow-diverting stents, double-layer micromesh stents, double-lumen balloons and microvascular plugs and are also associated with high rates of technical success.

**Conclusion:**

Complex techniques such as stent-assisted coiling and balloon-remodeling techniques are useful techniques and require advanced embolisation skills and are further described.

## Background

Abdominal visceral artery aneurysms (VAAs) are complex to manage but are increasingly the domain of the interventional radiologist. Developments in endovascular detachable coiling, liquid embolic agents and novel stent and stent-graft technology, now allow the safe treatment of more complex aneurysms. Visceral artery aneurysms by definition generally involve a pathological dilatation of branches of the coeliac, superior mesenteric, inferior mesenteric, or renal arteries. The aim of embolisation therapy is the prevention of rupture.

VAAs are classically subdivided into true and false aneurysms (Belli et al. 2012). A true aneurysm is a localized dilatation of the artery by more than 1.5 times the expected arterial diameter with involvement of all three layers of the arterial wall. True aneurysms may occur because of underlying arterial pathology such as atherosclerosis, fibromuscular dysplasia, and arteritis. True VAAs show a 0.1–2% prevalence, and most true VAAs are asymptomatic at diagnosis. They are often incidentally detected on cross-sectional imaging performed for other reasons. A minority may cause abdominal pain, which may be a harbinger of potential rupture (Tulsyan et al. 2007).

False aneurysms or pseudoaneurysms are effectively a contained arterial rupture that remains contained by adventitia or perivascular tissues. False aneurysms more commonly occur because of inflammation, infection, or trauma (Madhusudhan et al. 2016). Up to 70% of pseudoaneurysms and 20% of true aneurysms are liable to rupture and in this context, expected mortality can be unpredictable ranging from 25 to 100% (Pitton et al. 2015). There are further risk factors for rupture such as a hyperdynamic circulation, which may be exacerbated by pregnancy, portal hypertension and infections. Rupture is seen more commonly in hepatic, pancreatic and SMA aneurysms than renal and splenic artery aneurysms (Rijn et al. 2017).

### General indications for treatment of VAA

**Fig. 1:**
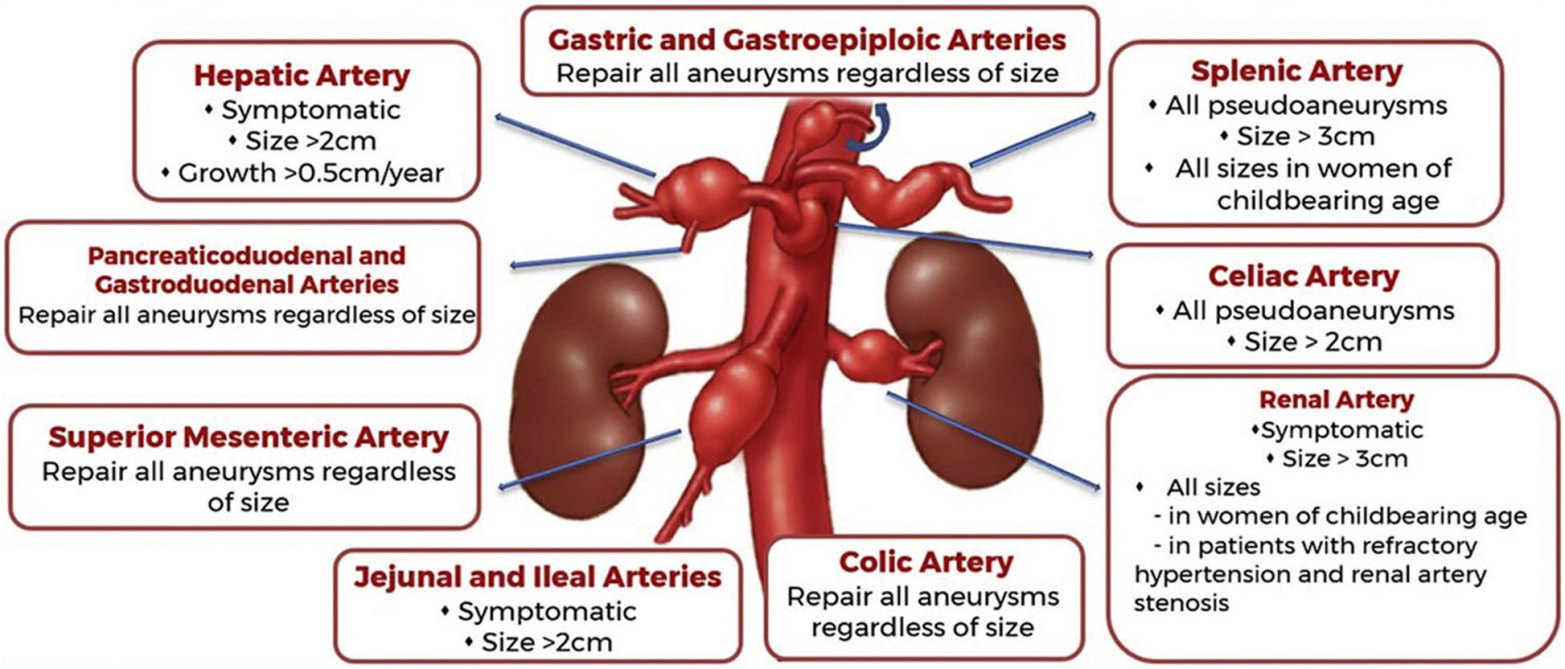
Diagram of Indications of Management of VAAs based on the Society of Vascular Surgery Clinical Practice Guidelines (Chaer et al. 2020)

The Society of Vascular Surgery (SVS) set guidelines to manage VAAs based on the affected artery, size of the lesion, rate of growth, the associated symptoms, and the potential pregnancy status (Chaer et al. 2020) (Fig. 1).

### Evolution of endovascular treatment in the management of VAAs

Throughout the last two decades, endovascular repair has increasingly become the first-line treatment (Martinelli et al. 2019). The choice between surgery and endovascular intervention has been mainly based on anatomical suitability and patient comorbidities (Gabelmann et al. 2002). The endovascular treatment of VAAs has also been the first option in the most challenging situations, such as abdominal sepsis or pancreatitis where surgical access may be precluded (Song et al. 2018).

Based on the morphology, endovascular treatment of VAAs is most readily suitable for saccular aneurysms with a narrow neck and aneurysms of vessels that are not the sole arterial inflow to that organ. An aneurysm is considered optimal for endovascular management if the front and back door vessels of the aneurysm can be accessed and occluded by a catheter-based system and if end organ perfusion can be preserved by collateral flow or stent graft therapy (Chadha and Ahuja 2009).

Aneurysm reperfusion is the most frequent cause of treatment failure after embolisation (Martinelli et al. 2019) and is a key consideration when selecting the most appropriate endovascular technique. In this review, we will describe different endovascular techniques available for treatment of true VAAs according to their anatomy and vascular territory.

### Endovascular strategy

When selecting the endovascular technique, many factors should be taken into consideration including the: site, size of the aneurysm as well as length, width, and shape of the aneurysm neck along with front door/back door arterial feeding arteries.

A key consideration when selecting an endovascular technique is whether a particular embolisation technique can protect vital branches and avoid inadvertent end-organ ischemia or visceral damage.

Computed tomography angiography (CTA) is the imaging method of choice (Horton et al. 2007). CTA defines the location, shape and aneurysm size, assessment of the aneurysm wall/contents, and the course and relationship to other vessels (Piasek et al. 2018). Its limitations are a relative lower sensitivity for smaller aneurysms (< 4 mm) and the need for a large bolus of contrast that may increase the risk of contrast-induced nephropathy (Computed Tomography Angiography - an overview | ScienceDirect Topics n.d.). Digital subtraction angiography (DSA) remains the gold standard for detecting stenoses and aneurysms in medium and large arteries and in diagnosing patients with vasculitis (Conventional Angiography - an overview | ScienceDirect Topics n.d.). DSA enables real-time assessment of the haemodynamics of the source vessel, identification of any collateral supply, and expendability of donor inflow artery (Image-Guided Interventions and - 3rd Edition n.d.), however its role is now preferentially therapeutic rather than diagnostic.

Excluding the aneurysm from the circulation and preservation of flow through the parent artery is the aim of VAA treatment. Regarding the selection of embolic agents, accurate evaluation of size and length of a stent-graft, the size, and length of coils/coil packing density, or embolic plugs, and estimation of the amount of liquid embolic agents are essential in formulating a management strategy.

Catheter access to the aneurysm including assessment of the take-off angle of the native artery, ostial stenosis and vessel tortuosity should be evaluated to decide via which arterial access approach is optimal (femoral, brachial, axillary, or radial) for successful exclusion of the aneurysm.

## Main text

### Endovascular techniques

#### Stent-graft placement

Placement of a stent-graft across an aneurysm involving a large-calibre vessel is at first sight, an attractive option because it will potentially exclude the aneurysm while maintaining patency of the parent vessel. Stent-grafts are however less commonly used for the treatment of VAAs (Dorigo et al. 2016; Spiliopoulos et al. 2012) often due to technical factors (Image-Guided Interventions and - 3rd Edition n.d.). Unfavourable anatomy of the visceral artery origin or arterial tortuosity or both (e.g. splenic artery) may lead to difficulty inserting a guiding sheath into a suitable position. The sheaths required for stent-graft placement are typically large-bore although there are now several stent-graft systems that allow placement of stents up to 11 mm via a 7 or 8 Fr sheath or guiding catheter. Embolisation of any non-essential branch arteries before stent placement may also be required to avoid any ongoing perfusion of the aneurysm post stent-graft placement. This technique may be also associated with a risk of distal organ ischemia (e.g. in renal or hepatic territories) related to the manipulation of the devices especially when inaccurate maneuvers are performed. After the procedure, a dual anti-platelet therapy for 6 months and lifelong aspirin (75 mg per day) should be given to prevent stent-graft thrombosis (Venturini et al. 2017).

When considering stent-graft insertion, key factors to consider are the diameter discrepancy between the two landing zones on either side of the aneurysm neck and whether there are any important branch or collateral arteries that arise either off the aneurysm or at any planned landing zones that may preclude placement of a stent-graft. Technical success rate of stent grafts may achieve 80–97% (Venturini et al. 2017).

#### Transcatheter arterial embolisation

Table 1 shows different endovascular transcatheter arterial embolisation techniques***.***

**Table 1:** Different endovascular transcatheter arterial embolisation techniques and their mode of action

**Parent artery preservation**	
Sac packing	Only the aneurysm sac is filled with the embolic material
Balloon remodeling or stent-assisted coiling	Balloon catheter is placed across the neck of the aneurysm and acts as a scaffold for coiling or liquid embolisation by the side of the inflated balloon Self-expanding bare stent is placed across the neck of the aneurysm and acts as a scaffold for coil embolisation through the interstices within the stent
Multi-layer flow diverting stents	They divert the blood through the vessel lumen with substantially reduced flow through their tight wire mesh away from the aneurysm sac
**Parent artery sacrifice**	
Trapping (sandwich, isolation, and front-to-back-door techniques): with or without sac packing	Embolic materials (coils or plugs) are deployed distally and proximally to the aneurysm neck
Inflow occlusion (proximal embolisation)	Occlusion proximal to the aneurysm neck

### Parent artery preservation

#### Sac packing with embolic material

Filling of the aneurysm sac with embolic material may be used as the sole method of exclusion of the aneurysm from the circulation. Sac packing is most optimal for saccular aneurysms with a narrow neck, which allows safe retention of embolic material within the sac maintaining the patency of the parent vessel (Madhusudhan et al. 2016). The embolic materials that are most commonly used are coils or liquid agents however coil packing of the aneurysm sac is often a most elegant technique and in many patients is the method of choice with packing density is a statistically significant predictor of complete occlusion, where technical success rate may reach 100% on a packing density of ≥ 24% in VAAs (Madhusudhan et al. 2016; Loffroy et al. 2008; Regus and Lang 2016; Kok et al. 2016; Guo et al. 2016; Yasumoto et al. 2013; Tosello et al. 2017).

This technique particularly with coils, should be classically avoided in pseudoaneurysms, because it may cause expansion of the sac with increasing the risk of its rupture owing to absence of the three-layered wall and being contained only by surrounding haematoma. Although it may initially result in a satisfactory angiographic outcome, with sac thrombosis and preserved patency of the parent vessel, subsequent lysis of the surrounding haematoma will almost invariably cause the “coil ball” to fall away from the arterial defect, and the pseudoaneurysm will then recanalize and re-bleed. In some situations, coil packing may also be used for pseudoaneurysms if there is no other choice (Loffroy et al. 2010). To avoid late coil compaction or recanalization, tight coil packing of saccular VAAs, with packing density of at least 24%, is essential. Precise measurement of three-dimensional aneurysm size and intraprocedural calculation of packing density are essential to ensure adequate coil packing. Denser coils and adjunctive neck remodeling techniques should be considered especially in large and wide-neck aneurysms (Yasumoto et al. 2013).

#### Balloon remodelling or stent-assisted coiling

**Fig. 2:**
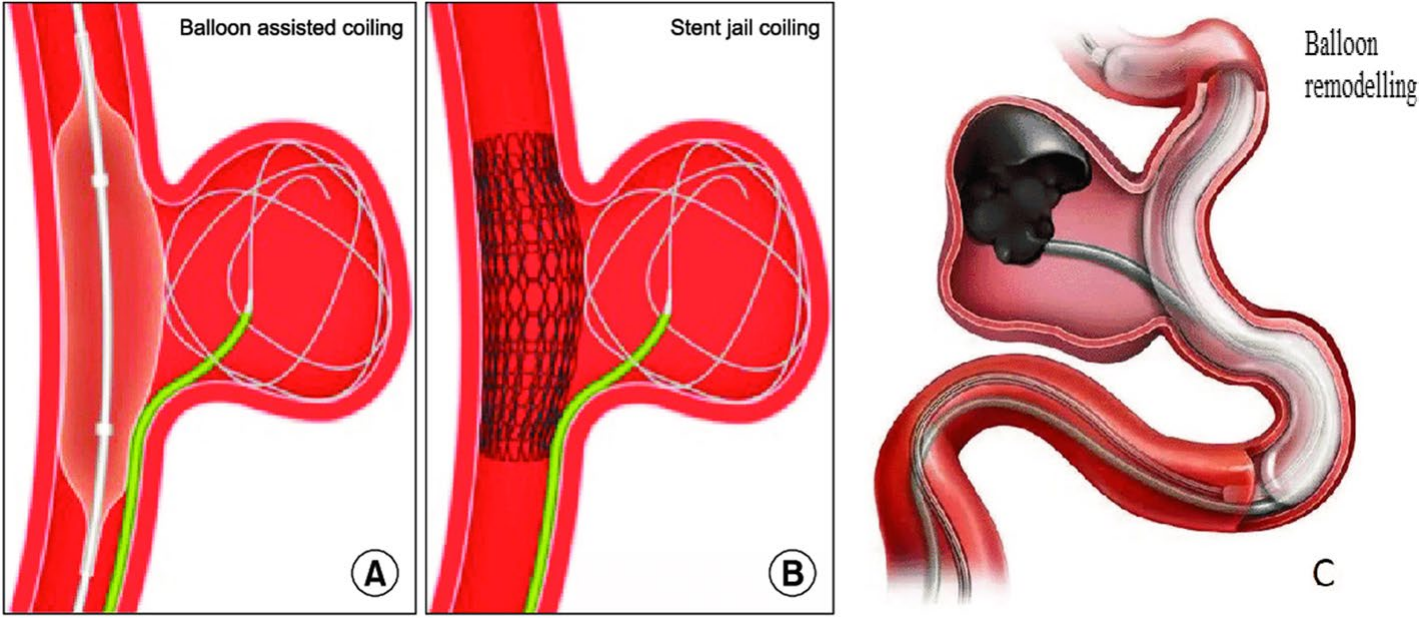
Diagram of stent-assisted coiling and balloon remodelling

These two techniques (Fig. 2) are helpful to protect the visceral arterial lumen when a covered stent cannot be used (Centenera et al. 1998; Favelier et al. 2012), with excellent technical success reaching 100%, and a low rate of major complications (Secco et al. 2021).

Stent-assisted coiling technique may be useful in certain situations where anatomy precludes coil packing of the sac alone. It is a combination of an uncovered stent placed across the neck of the aneurysm and packing the aneurysm sac with coils via a microcatheter introduced through the stent mesh (or placed within the sac (“jailed”) before deployment of the stent). The stent serves as a mechanical scaffold for safe placement of coils, preventing their herniation into the parent artery and enabling dense coil packing (Irie et al. 2003; Li et al. 2014).

In the balloon remodeling technique, a non-detachable balloon is temporarily inflated across the neck of the aneurysm during coil packing or liquid embolic injection. At the end of the procedure, the balloon is removed, and no device is left in place in the parent vessel. The aim of this technique is to form a cast within the aneurysm and once the balloon is deflated, flow is preserved into the distal branch vessels after exclusion of the aneurysm from the circulation.

In saccular aneurysms, the balloon is simply placed in the parent vessel across the aneurysm neck. In bifurcation aneurysms, there are several options (Song et al. 2018). One balloon may be placed within the parent vessel and the bifurcation artery. The balloon is inflated sufficiently to completely cover the neck of the vessel that the operator wishes to preserve. Another method is to place two balloons in front of the aneurysm neck. Moreover, a double lumen microcatheter remodeling balloon could be placed in front of the neck with a micro guidewire stabilizing access inside the neck. Coils or liquid embolic agents e.g., Onyx (Medtronic Inc.) can then be injected into the aneurysm via the second lumen.

#### Multi-layer flow diverting stents

Flow diverting stents have been used effectively and safely for the management of intracranial aneurysms, especially for large and wide-necked aneurysms that are not amenable to conventional endovascular treatment with coiling. The theory behind the use of these devices is that they enhance thrombosis of the aneurysm sac by diverting blood through the vessel lumen with substantially reduced flow through the tight wire mesh into the aneurysm sac (Dholakia et al. 2017). In neurovascular practice, there are several flow diverting stents available including the Pipeline Embolisation Device (PED, Medtronic, MN, USA), SILK flow diverter (Balt Extrusion, Montmorency, France), the Flow Reduction Endoluminal Device (FRED, Microvention, CA, USA), p64 (Phenox, Bochum, Germany), LEO Baby (Balt Extrusion, Montmorency, France), and Derivo Embolisation Device (Acandis GmbH & Co. KG, Pforzheim, Germany). The Derivo Embolization Device (DED) is a second-generation flow diverting stent with a novel surface finishing that might lead to reduced friction and low thrombogenicity in the treatment of intracranial aneurysms. These devices have been reported to be effective in the management of large or giant complex aneurysms such as those with a wide neck or fusiform appearance. More recently, the Surpass Streamline device, a self-expandable braided flow diverting stent composed of a cobalt-chromium alloy has been shown to effectively treat such aneurysms with 98% technical success and high occlusion rates at follow-up (Wakhloo et al. 2015). Although there are several advocates for their efficacy, they are not widely used in the treatment of VAAs because of a lack of good quality published evidence.

### Parent artery sacrifice

#### Trapping ‘Sandwich Occlusion’

This technique is most easily performed by placing metallic coils or vascular plugs on either side of the aneurysm and has been used more often than other techniques. When performed correctly, will be curative with little risk of recurrence. This technique has clinical success rates of > 90% (Spiliopoulos et al. 2012). The most common plug used for treating VAAs are the Amplatzer vascular device (Abbott). The currently available Amplatzer devices are the AVP, AVP II, AVP III, and AVP IV (Leyon et al. 2014). Its mechanisms of action are similar to coils whereby the device “plugs” the vessel wall thereby contacting the vessel wall and releasing thromboxane thus promotes thrombogenesis. The device can be retrieved back into the delivery catheter, repositioned, and redeployed soon after placement, if the site of initial placement is thought to be inadequate. The AVP may be appropriate for short vessel landing zones. AVP II is designed to reduce the time to occlusion and better conforms to vessel landing zones, whereby AVP III provides most rapid occlusion of all AVP devices and is ideal for high flow situations. The AVP IV is flexible, can be negotiated through tortuous anatomy and can be delivered through a 4- or 5-Fr 0.038″ compatible catheter.

It is essential that complete occlusion of the vessel beyond the aneurysm be achieved before proximal embolisation is performed so that the possibility of recanalisation via collaterals is prevented. Clearly, if bleeding were to recur via this route because of inadequate distal embolisation, the chance of successfully re-treating the aneurysm will be markedly reduced.

#### Inflow occlusion ‘Proximal embolisation’

This technique consists of embolic occlusion of the feeding artery from which the aneurysm arises. Generally, it is used for distal lesions in intraparenchymal vessels, and visceral artery aneurysms with a good rate of collateralization, as splenic aneurysms, through selective embolisation of the feeding vessel using a variety of embolic agents such as microcoils or liquids. This may result in a small area of peripheral parenchymal infarction, mostly without any clinical consequences as long as the embolised vessel is small and does not supply a large territory, and the organ has a good rate of collateralization. The risk of recanalisation after embolisation with liquids seems to be rare in comparison with other embolic agents (Madhusudhan et al. 2016; Dorigo et al. 2016; Spiliopoulos et al. 2012; Loffroy et al. 2008; Regus and Lang 2016; Kok et al. 2016; Guo et al. 2016; Hemp and Sabri 2015) with 100% technical success rate, and 96.4% clinical success rate (Omar et al. 2021).

#### More recent developments

##### Double-layer micromesh stents

This is a recent development and consists of a retrievable, self-expanding outer nitinol stent cased an internal micromesh layer. The stent is an open cell highly flexible device, which allows it to adapt to tortuous anatomy, while the micromesh allows thrombosis of the aneurysm via flow diversion (Roadsaver ®, Terumo, Tokyo, Japan). The device is also repositionable and retrievable. This stent has been used in the treatment of stenotic carotid lesions, mainly for complex atherosclerotic plaques with a low complication rate of 3% (Machnik et al. 2020), however its use in visceral and peripheral vessels remains off-label (Akkan et al. 2017).

##### Double-lumen balloons

The use of double-lumen balloons has been mainly reported in neurointerventions and has recently been reported for use in the treatment of VAAs. This technique offers balloon-assisted coil embolisation with an option to deploy a stent across the aneurysm neck if the coils appear at risk of migration when the balloon is deflated (e.g. Ascent Dual lumen balloon, DePuy Synthes) (Onal et al. 2020).

##### Microvascular plugs

Since being available in 2013, they have mainly been used in neurointerventions and remain new to the endovascular management of VAAs. These plugs (MVP MicroVascular Plug System, Medtronic) are nitinol, cage-like design scaffold with a polytetrafluoroethylene cover on one side and a preloaded detachable pusher (Beaty et al. 2015). There are case reports of use in visceral arteries for trauma, tumour embolisation, and aneurysm embolisation.

### Specific true visceral artery aneurysms

#### Renal Artery Aneurysms (RAAs)

**Fig. 3:**
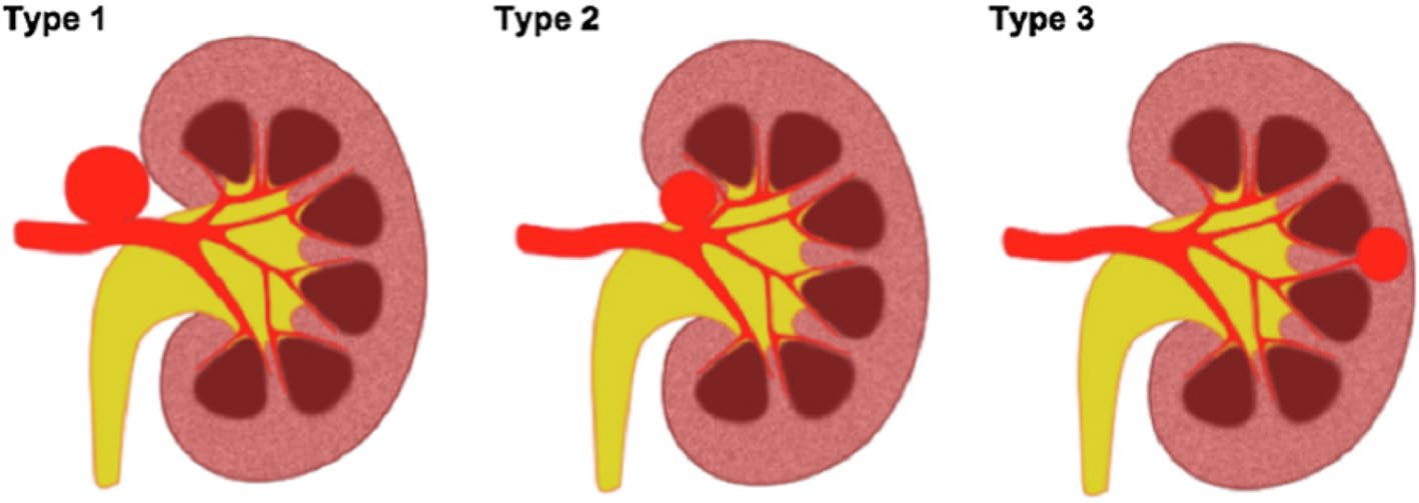
Diagram of the Rundback Classification (Chung et al. 2016)

**Fig. 4:**
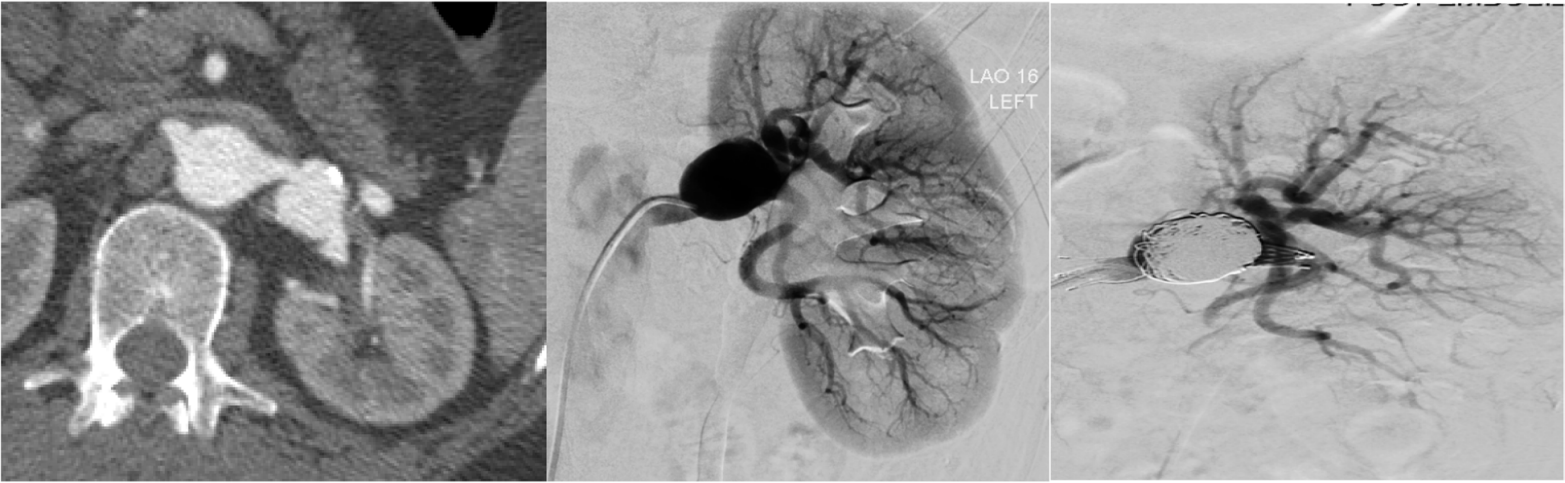
Left renal artery aneurysm (Rundback type 2) managed by stent assisted coiling

RAAs represent 22% of VAAs and treatment of RAAs is generally recommended at 20 mm, in symptomatic patients and of any size in female patients of childbearing age. Complexity of treatment is predominantly dependent on the aneurysm location (Kashef and Hamady n.d.). Based on the Rundback classification (Fig. 3), a type 1 RAA, arising from the main trunk, can be treated with covered stents with a landing zone of 15 mm on either side deemed desirable. A type 2 RAA is often situated at the extrarenal proximal arterial bifurcation, and type 3 RAAs arise from the distal branches (Rundback et al. 2000). Due to the necessity to preserve as much renal parenchyma as possible, type 2 RAA that arise at the extra renal proximal arterial bifurcation, should be treated by stent-assisted coil embolisation (Fig. 4) or the balloon remodeling technique with a non-adhesive liquid embolic agent (e.g. Onyx (Medtronic, Santa Rosa, CA)) with or without coils (Chung et al. 2016).

#### Splenic Artery Aneurysms (SAAs)

**Fig. 5:**
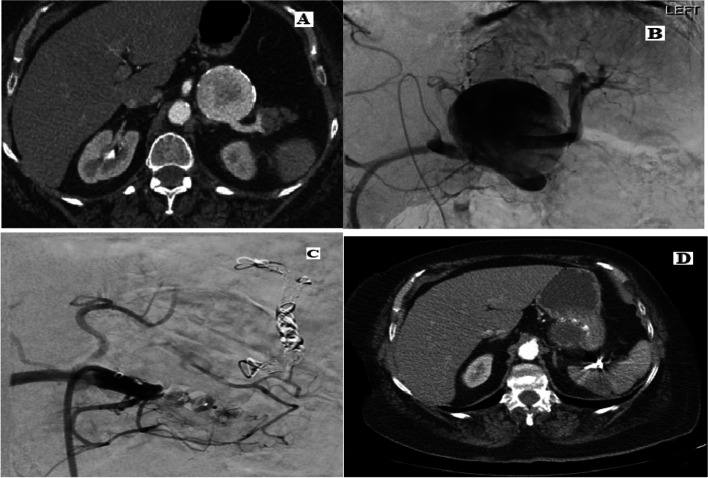
Splenic artery aneurysm managed by front and back door embolisation using vascular plug for the front door and coils for the back door. **A** Axial arterial phase post contrast CT showing 5.5 cm main artery SAA. **B** Coeliac angiogram confirms the SAA. **C** Embolisation using vascular plug for the front door and coils for the back door. **D** 2 months follow up arterial phase post contrast CT confirming exclusion of the aneurysm from the circulation with less than 20% infarction of the splenic parenchyma without clinical significance

SAAs account for 60% of all VAAs. Treatment of SAAs is generally recommended at a size threshold of 30 mm, in symptomatic patients and of any size in women of childbearing age because of the risk of rupture (Chaer et al. 2020). It is beneficial to preserve the parent artery to avoid end-organ ischemia but not crucial given this technique in the management of splenic trauma, where proximal splenic artery embolisation, between the dorsal pancreatic artery and great pancreatic artery, is performed in cases of multifocal injury, whereas distal embolization is reserved for cases of focal vascular injury. There is no significant difference in efficacy of splenic salvage between proximal and distal embolization (Quencer and Smith 2019). Another advantage of proximal embolisation over distal embolization is faster procedure times, which is important in trauma patients whose hemodynamic stability can change quickly. Aneurysms involving the main splenic artery are best treated by embolisation using coils,preferably 0.035′′ coils to 0.018′′ coils given their higher radial strength (Fig. 5), or Amplatzer plugs. Stent grafts are generally not feasible due to severe tortuosity of the vessels. Distal embolisation of SAAs involving the intrasplenic arteries is best managed by super-selective embolisation using particles, glue (such as N-butyl cyanoacrylate), gel foam and/or coils (Quencer and Smith 2019).

#### Hepatic Artery Aneurysms (HAAs)

HAAs are the second most common true VAA. HAAs are more predominant in men and tend to be extrahepatic in 60% of cases. Treatment of HAAs is generally recommended at a size threshold of 20 mm, in symptomatic patients and in female patients of childbearing age. HAAs are commonly associated with hypertension (in up to 72% of patients). Up to 31% of patients with HAA have VAAs at other sites, but most HAAs are solitary and occur due to atherosclerosis (Belli et al. 2012). It is mandatory to confirm the patency of the portal vein if extensive intrahepatic arterial embolisation or embolisation of the proper hepatic artery is to be undertaken. In most patients, the main endovascular option involves coil embolisation of the afferent and efferent arteries on either side of the HAA.

**Fig. 6:**
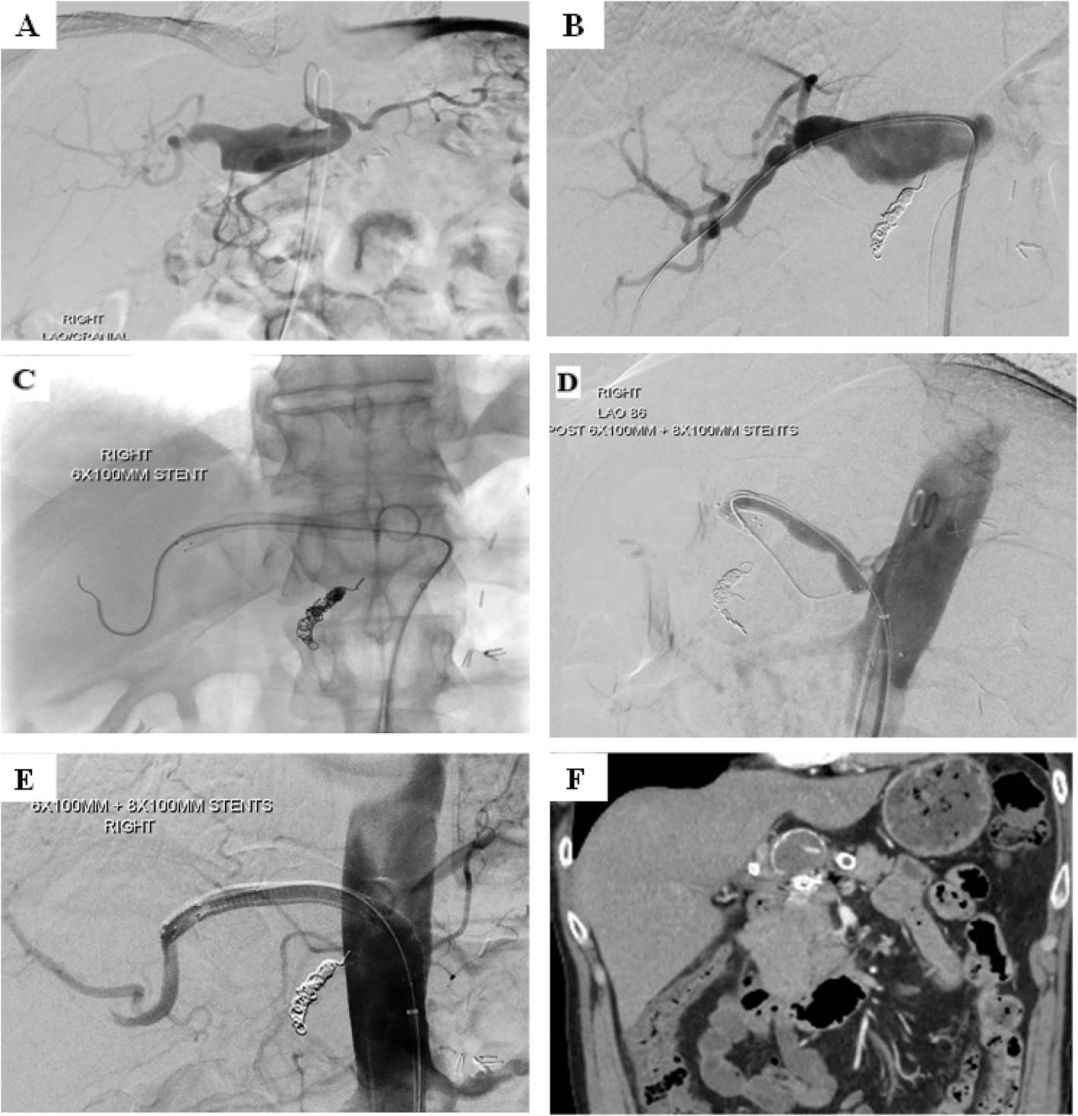
Management of a true CHA aneurysm with stent-grafts and GDA embolisation. **A** Angiography confirmed the presence of a large aneurysm arising from the proximal proper hepatic artery extending to the bifurcation of the proper hepatic artery. **B** The gastroduodenal artery arising from the proximal aspect of the aneurysm was embolised with coils. **C** and **D** Two Viabahn stent grafts were placed extending from the right hepatic artery proximally to the origin of the coeliac trunk and completion angiography showed an excellent result with exclusion of the hepatic artery aneurysm. **E** Complete aneurysmal exclusion from the circulation on final angiogram. **F** On the 30 month follow up, the hepatic artery stents appeared occluded, however there are hepatic collaterals noted from the left gastric artery with patent portal vein. The hepatic artery aneurysm remains occluded

A covered stent is a valuable option in the management of HAA arising from the common HA (Zhakubayev et al. 2018) (Fig. 6). Flow-diverting stents may be of value in maintaining patency of adjacent arteries, e.g., a HAA arising from the common HA involving the common hepatic origin, the gastroduodenal artery, and the hepatic bifurcation (Balderi et al. 2010). Although outcomes data are limited, open repair and endovascular treatment have comparable outcomes, with a slightly higher rate of reintervention in the endovascular treatment group (Barrionuevo et al. 2019).

#### SMA (SMAAs), Gastroduodenal (GDA) and Pancreaticoduodenal (PDA) aneurysms

Generally, these aneurysms are rare. SMAAs usually involve the most proximal 5 cm of the SMA (Pilleul and Beuf 2004 Nov). Underlying etiologies include atherosclerosis, collagen vascular disease, cystic medial dysplasia, polyarteritis nodosa, and infection (Stone et al. 2002). A small proportion of PDA aneurysms are indirectly caused by compression by the median arcuate ligament, as a result of a high flow state in the pancreatic arterial arcade due to stenosis of the celiac artery (Chivot et al. 2016). On the other hand, pseudoaneurysms of the GDA and PDA are much more common and usually occur secondary to inflammatory or infectious processes (e.g. acute pancreatitis). These aneurysms should be treated regardless of their sizes. Selection of the optimal management technique depends on the proximity of important side branches requiring preservation of patency. In SMAAs, hybrid procedures involving the insertion of bypass grafts before embolisation can increase the role of endovascular techniques in such complex territories.

**Fig. 7:**
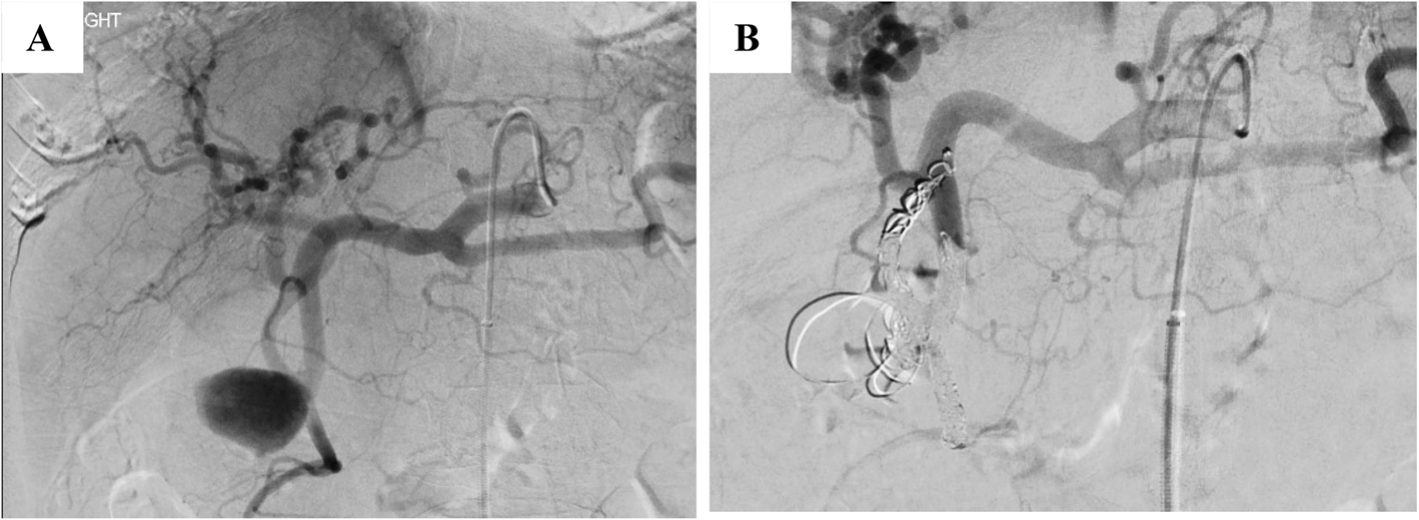
Embolisation of GDA aneurysm by sandwich occlusion technique with micro coils. **A** GDA aneurysm on selective angiogram of the celiac axis with associated replaced right hepatic artery arising from the GDA at the neck of the aneurysm. **B** Embolisation of the front and back doors of the GDA aneurysm as well as the replaced right hepatic artery using 4, 5 and 6 mm detachable 0.018 coils

In GDA & PDAs the parent artery can usually be sacrificed because of the rich collateral circulation. It is necessary to ensure that retrograde perfusion of the aneurysm cannot occur from collateral vessels (e.g., inferior pancreaticoduodenal artery). In general treatment of these aneurysms is mainly performed through a front and back door closure using microcoils for selective placement of embolic material in these small, often tortuous arteries (Fig. 7). Previous surgery or radiotherapy may increase the potential for bowel ischemia and infarction as collateralisation may be reduced. An occluded origin of either the CAA or SMA makes embolisation of GDA and PDA much more complex and in these cases, maintaining patency of the parent artery is mandatory.

## Conclusions

True VAAs are uncommon but have the potential for rupture and death. Management is based on accurate assessment of their vascular anatomy and the selection of the optimal technique to achieve exclusion of the aneurysm while preserving important branches. Interventional radiologists should know the choice of endovascular techniques and endovascular devices available to safely treat these lesions.

## Data Availability

No specific datasets were required for this review article.

## Abbreviations

VAAs: Visceral artery aneurysms
SMA: Superior mesenteric artery
CHA: Common hepatic artery
CTA: Computed tomography angiography
MR: Magnetic resonance
DSA: Digital subtraction angiography
RAAs: Renal artery aneurysms
SAAs: Splenic artery aneurysms
HAAs: Hepatic artery aneurysms
CAAs: Celiac artery aneurysms
SMAAs: Superior mesenteric artery aneurysms
GDA: Gastroduodenal aneurysms
PDA: Pancreaticoduodenal aneurysms
